# YouTube Videos: A Learning Tool for Periodontology Education

**DOI:** 10.7759/cureus.76049

**Published:** 2024-12-19

**Authors:** Saumya Singh, Girish Suragimath, Siddhartha Varma, Sameer A Zope, Vaishali S Mashalkar, Apurva V Kale, Ashwinirani SR

**Affiliations:** 1 Department of Periodontology, School of Dental Sciences, Krishna Vishwa Vidyapeeth (Deemed to be University), Karad, IND; 2 Department of Oral Medicine and Radiology, School of Dental Sciences, Krishna Vishwa Vidyapeeth (Deemed to be University), Karad, IND

**Keywords:** dental education, dental students, periodontology, social media, youtube

## Abstract

Introduction

Video-based learning has proved to be an effective tool for education and knowledge gain in all fields. YouTube is a free video-sharing website consisting of numerous educational and skill development videos; you can even create and upload your videos to share knowledge and experience with others. YouTube contains videos about simple button sewing to space rocket preparation. YouTube also contains dental education videos, particularly in the field of periodontology.

Aim

This study aimed to assess the effectiveness of YouTube videos as an educational tool in periodontology and evaluate the knowledge gained from these videos through a structured questionnaire. This investigation aimed to provide YouTube's potential as a supplementary resource for enhancing periodontology education.

Material and methods

This experimental, descriptive questionnaire-based study was conducted among 100 final-year dental undergraduate students. Student feedback and responses were gathered through a closed-ended structured questionnaire. A pre-test consisting of 30 questions was administered to assess students' knowledge before watching the videos. YouTube videos on periodontology topics, such as bone grafts, dental implants, and periodontal flaps, were circulated among selected students. The students were instructed to watch the selected videos, followed by a post-test with the same questions to evaluate their knowledge gain. The pre- and post-test responses were analyzed using a paired t-test in Statistical Product and Service Solutions (SPSS, version 24; IBM SPSS Statistics for Windows, Armonk, NY) software.

Results

The mean score of the students in the pre-test was 6.74 ± 11.53, and that in the post-test was 25.64 ± 8.68, so the difference in the mean score of the pre-and post-tests was 18.90 ± 14.45 after watching YouTube videos on periodontology topics with a P-value (<0.0001), which shows significant knowledge gain. The majority of students (N = 80-90, 80-90%) showed interest in using YouTube videos for periodontology education.

Conclusion

This study demonstrates that YouTube videos are highly effective and well-received adjunctive educational tools for dental students, particularly in the field of periodontology. The high levels of satisfaction, knowledge gain, and engagement observed in this study suggest that integrating YouTube videos into the curriculum can significantly enhance the learning experience.

## Introduction

Dental education has traditionally relied on textbooks, lectures, and clinical demonstrations to impart foundational knowledge and skills to students. While these methods are indispensable for building a strong theoretical base, they often present limitations [1]. Conventional teaching methods may not adequately address the diverse learning styles of students, and static representations, such as images or text, can struggle to convey complex procedures effectively [2].

Dental education has undergone significant transformations, emphasizing critical thinking, problem-solving, and student-centered learning approaches [3]. These pedagogical shifts aim to engage students more actively and foster deeper understanding. Dental skills training is a very important part of preclinical learning in dental education and has a long history [4]. There are different social media platforms, such as Facebook, Twitter, YouTube, and Instagram. Nowadays, college students use social media on a daily basis [5].

YouTube, founded in 2005, is the largest and most popular video-hosting platform and has emerged as a potentially transformative tool in this context [6]. It provides free access to a vast array of video-based content, and it has increasingly become a resource for patients seeking health information and students pursuing supplementary educational material [7].

Traditionally, information about medicine and dentistry was accessed through direct consultation with experts or formal education settings. However, the proliferation of internet use, even in developing countries, has made online resources, including YouTube, a convenient and accessible option for learning [8]. Unlike traditional lectures and live demonstrations, which are inherently limited by their one-time nature, video content allows for repeated viewing. This feature can be particularly advantageous for students needing additional exposure to complex concepts or procedures to enhance understanding and retention [9].

Dental students recommended YouTube as an educational resource and expressed interest in having video tutorials created by their dental school faculty uploaded to YouTube or other social media platforms [10]. Given the growing integration of video-based learning in dental education, understanding its impact on student learning outcomes is essential. This study aimed to evaluate the effectiveness of YouTube videos in periodontology education and compare the knowledge gained through these videos using a structured questionnaire.

## Materials and methods

This experimental, descriptive questionnaire-based study was conducted among final-year BDS students from the School of Dental Sciences, Karad, Maharashtra, India. This study aimed to evaluate the effectiveness of YouTube videos in periodontology education and compare the knowledge gained through these videos using a structured questionnaire.

YouTube video selection

Periodontal faculty selected the videos based on their quality, clarity, views, and likes. Opinions of senior subject experts were included for the finalization of the videos.

Inclusion criteria for video selection: Videos with evidence-based content; clear and accurate demonstrations of periodontal procedures; professionally produced videos (e.g., academic or institutional sources); and content aligned with the study’s educational goals and syllabus.

Exclusion criteria for video selection: Videos with inaccurate or non-evidence-based information; poorly produced or low-resolution content; content created by non-verified or non-professional sources; and videos with excessive commercial bias or promotional material.

Questionnaire development

The questionnaire was fabricated by referring to the relevant literature and textbooks on the topics of bone grafts, dental implants, and periodontal flaps. A pilot study was conducted among 30 final-year BDS students. YouTube videos on bone grafts were circulated among selected students to authenticate the validity of the questionnaire. The internal consistency of the responses in our structured questionnaire was calculated using Cronbach’s alpha formula. The test-retest reliability of the questionnaire was tested by administering the questionnaire to the smaller pilot group at two different points under similar conditions, and the results were compared using the correlation coefficient to assess the stability of the responses over time. The reliability indicator/score was found to be more than 0.70 in the present study. The items scale content validity index was also calculated based on the expert judgment method by consulting domain experts in periodontology to review the questionnaire. The questionnaire, printed in English, consisted of 10 questions on each topic, such as bone grafts, dental implants, and periodontal flaps. The first 10 questions were on bone grafts, followed by 10 on dental implants, and the last 10 on periodontal flaps. The questionnaire is included in the appendices section (Table 4) of the article.

Ethical issue

Ethical clearance was obtained from the university’s ethics committee of Krishna Vishwa Vidyapeeth (KVV), Deemed to be University (KVV/IEC/10/2024, dated 03rd August 2024) before commencing the study.

Study design

The pre- and post-test questionnaires were circulated through Google Forms, and no details such as name, email address, or WhatsApp number were collected in the Google Forms to maintain anonymity. Informed consent was obtained from all the students before they enrolled in the study. Google Forms, which consisted of 30 closed-ended questions, was the means to acquire data from students willing to be part of the study.

Sample size estimation

The total sample size of 100 was obtained based on the level of significance (alpha error of 5% and power of 80%) and statistical formula using n = (Z1)2 [P(1-P)]/d2.

Data collection

The data obtained were entered into an Excel sheet, and the results were analyzed using a statistical package of social studies (Statistical Product and Service Solutions (SPSS, version 24; IBM SPSS Statistics for Windows, Armonk, NY).

Statistical analysis

The responses were compiled using a Microsoft Excel sheet (Microsoft® Corp., Redmond, WA). A statistical analysis was carried out using paired t-tests with the help of SPSS software.

## Results

The study aimed to assess the use of YouTube videos for periodontology education and compare the knowledge gained using YouTube videos for periodontology education through a questionnaire. A hundred students (62 females and 38 males) aged between 20 and 23 years participated in the study (Table 1).

**Table 1:** Demographic characteristics of the participants.

Characteristic	Details
Total Participants	100
Gender	Female: 62 (62%)
	Male: 38 (38%)
Age Range	20 to 23 years

**Figure 1:**
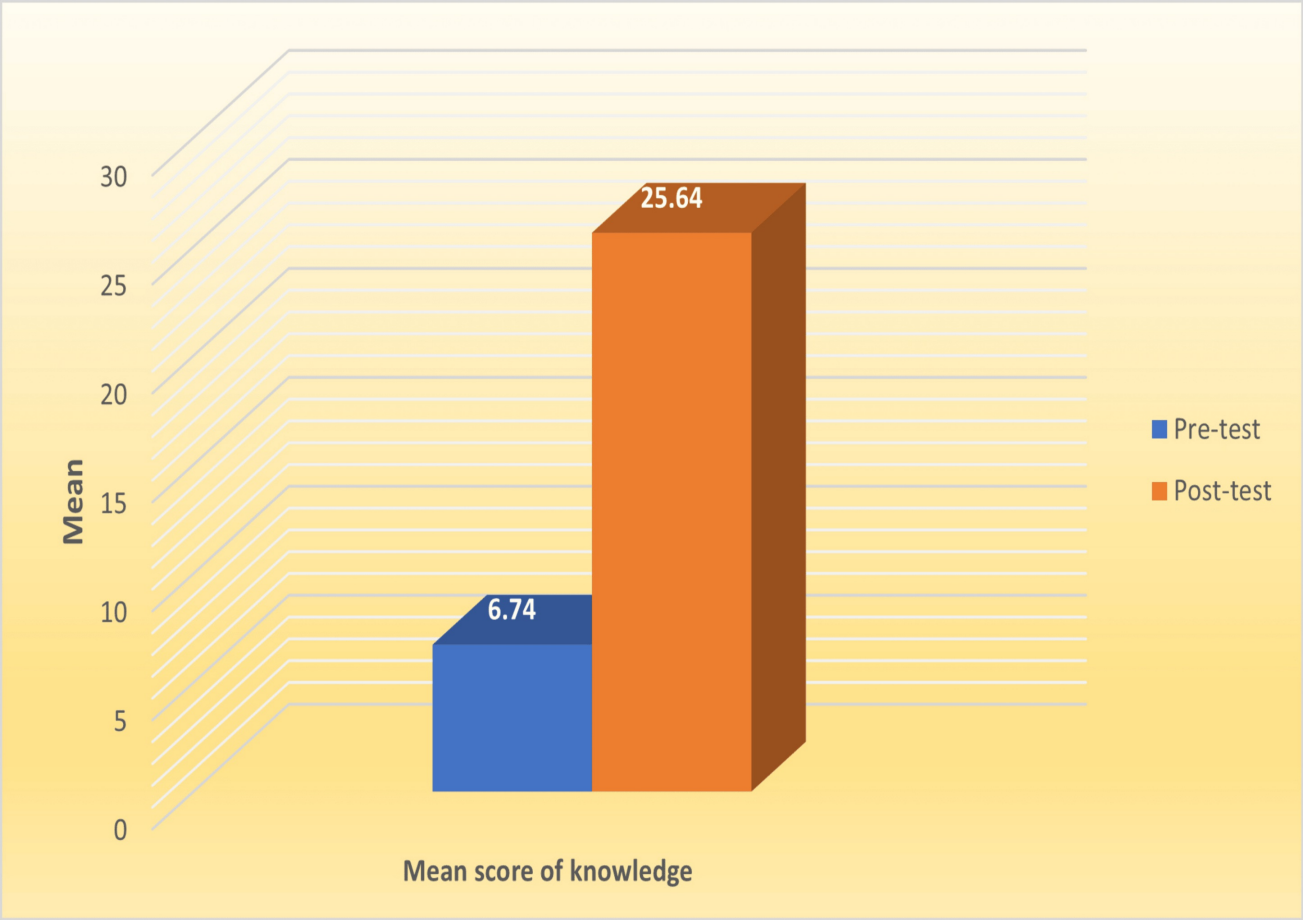
Knowledge gained among students from YouTube videos pre- and post-tests.

A paired t-test was used to analyze the data. The mean score of the students in the pre-test was 6.74 ± 11.53, and that in the post-test was 25.64 ± 8.68, so the difference in the mean score of the pre- and post-tests was 18.90 ± 14.45 with a P-value (<0.0001), which shows significant knowledge gain (Figure 1).

In the pre-test, participants did not score very high, with correct answers ranging between 12% and 32%. However, after the intervention, there was a significant improvement in their performance. Following the intervention, the percentage of correct answers increased significantly for each question, with post-test scores ranging from 68% to 96% (P-value <0.0001) (Table 2).

**Table 2:** Comparison of the question-wise assessment of pre- and post-tests among students.

Questions	Pre-test N = 100 (%)	Post-test N = 100 (%)	P-value
Q1	18 (18.00)	88 (88.00)	<0.0001
Q2	24 (24.00)	76 (76.00)	<0.0001
Q3	20 (20.00)	80 (80.00)	<0.0001
Q4	18 (18.00)	82 (82.00)	<0.0001
Q5	30 (30.00)	90 (90.00)	<0.0001
Q6	22 (22.00)	78 (78.00)	<0.0001
Q7	26 (26.00)	84 (84.00)	<0.0001
Q8	28 (28.00)	94 (94.00)	<0.0001
Q9	14 (14.00)	76 (76.00)	<0.0001
Q10	22 (22.00)	88 (88.00)	<0.0001
Q11	26 (26.00)	92 (92.00)	<0.0001
Q12	24 (24.00)	74 (74.00)	<0.0001
Q13	12 (12.00)	68 (68.00)	<0.0001
Q14	32 (32.00)	96 (96.00)	<0.0001
Q15	28 (28.00)	94 (94.00)	<0.0001
Q16	26 (26.00)	92 (92.00)	<0.0001
Q17	22 (22.00)	86 (86.00)	<0.0001
Q18	22 (22.00)	88 (88.00)	<0.0001
Q19	28 (28.00)	96 (96.00)	<0.0001
Q20	26 (26.00)	94 (94.00)	<0.0001
Q21	22 (22.00)	82 (82.00)	<0.0001
Q22	28 (28.00)	94 (94.00)	<0.0001
Q23	18 (18.00)	78 (78.00)	<0.0001
Q24	20 (20.00)	84 (84.00)	<0.0001
Q25	22 (22.00)	90 (90.00)	<0.0001
Q26	16 (16.00)	80 (80.00)	<0.0001
Q27	14 (14.00)	78 (78.00)	<0.0001
Q28	20 (20.00)	86 (86.00)	<0.0001
Q29	22 (22.00)	86 (86.00)	<0.0001
Q30	26 (26.00)	90 (90.00)	<0.0001

A majority of students showed a strong inclination towards the use of YouTube for future studies. A total of 82 (82%) students favor the use of YouTube for continued learning. A significant portion (N=90, 90%) of students found YouTube to be beneficial in reducing learning barriers and increasing their interest in the subject matter. Students expressed high satisfaction with using YouTube as a learning tool (N=94, 94%). There were no negative responses, underscoring the general acceptance and satisfaction with YouTube as a learning platform. In response to the question regarding the use of YouTube videos for exam preparation, 65 (65%) of students indicated they were "very likely," while an additional 16 (16%) reported being "likely" to utilize this resource. The majority of students (N= 80-90, 80-90%) showed interest in using YouTube videos for periodontology education (Table 3). The response rate for this study was 100%, with all 100 invited students completing both the pre- and post-test questionnaires.

**Table 3:** Perception of students towards YouTube videos.

Perception	Very likely N (%)	Likely N (%)	Neutral N (%)	Unlikely N (%)	Very Unlikely N (%)
I will apply it for further studying	40 (40%)	42 (42%)	04 (4%)	14 (14%)	00 (0)
YouTube videos helped me reduce the barriers and enhance my interest in learning	22 (22%)	68 (68%)	04 (4%)	06 (6%)	00 (0)
I was satisfied with using YouTube videos to learn	48 (48%)	46 (46%)	06 (6%)	00 (0)	00 (0)
I will use YouTube videos while preparing for exams	65 (65%)	16 (16%)	02 (2%)	17 (17%)	00 (0)

## Discussion

This study highlights the significant potential of YouTube as a learning tool in dental education, particularly in periodontology. The findings demonstrate a marked improvement in students' knowledge, with a post-test mean score of 25.64 ± 8.68, significantly higher than the pre-test mean scores of 6.74 ± 11.53, underscoring the platform’s effectiveness in enhancing understanding and retention of complex topics. This improvement is particularly important in fields such as periodontology, where visual and dynamic representations of clinical procedures are essential for mastering concepts such as bone grafts, dental implants, and periodontal flaps.

The results of our study align with findings from previous research. Hakami et al. demonstrated that video-based learning significantly improved students’ conceptual understanding and technical skills in orthodontics compared to traditional methods [11]. Similarly, our study found that (N=94, 94%) of students expressed satisfaction with YouTube as a learning tool, indicating its strong appeal as an engaging and effective medium. By using visual aids and interactive formats, YouTube overcomes many of the barriers associated with traditional learning, making it easier for students to grasp difficult topics. Burns et al. concluded in their study that 95% of respondents considered YouTube videos on clinical procedures to be a helpful learning tool, and 89% would like their dental school to post tutorials on YouTube, which was similar to our study [10].

As identified in this study, one of the key benefits of YouTube is its ability to reduce learning barriers. A total of 90 (90%) students agreed that YouTube videos enhanced their interest in learning and helped them overcome challenges in understanding periodontology concepts. This suggests that YouTube not only serves as an informational tool but also fosters motivation and engagement among students. Engaging students in learning is critical, particularly in technical fields such as dentistry, where understanding complex topics is essential.

Another important finding is that 79 (79%) students reported that they were very likely to use YouTube for exam preparation. This highlights the platform's practical value as a supplementary resource during assessments. The vast array of educational content available on YouTube allows students to reinforce their learning, review procedures, and clarify doubts. However, it is worth noting that 18 (18%) students were hesitant to use YouTube for exam preparation, which may be due to concerns over the credibility and reliability of some videos. This issue underscores one of the main challenges with YouTube as an educational tool: the quality of content can vary widely.

Concerns about the reliability of YouTube content are consistent with findings by Uzel et al., who evaluated pediatric dentistry videos and found that many lacked evidence-based information [12]. Non-academic sources were particularly noted for their lower quality and educational value. Similarly, this study emphasizes the need for academic institutions to play an active role in curating high-quality, evidence-based videos. By creating structured and reliable content, educational institutions can address students' concerns about accuracy while ensuring that the information aligns with established academic standards.

The preference for YouTube among students also reflects a broader trend toward digital learning tools. In this study, 82 (82%) students indicated they would likely continue using YouTube for further studies, demonstrating the platform’s strong appeal as a modern educational resource. The popularity of YouTube as a learning tool can be attributed to its accessibility, ease of use, and engaging formats. As Rajeh et al. noted, social media platforms such as YouTube can effectively support dental education when used appropriately, offering both students and educators new opportunities for learning [13]. Duncan et al. concluded that lecturers should take a more active role in recommending high-quality YouTube videos as supplementary learning resources, ensuring they thoroughly evaluate the content for accuracy and educational value before sharing it with students [14]. Patano et al. in his study demonstrated that e-learning is as effective as traditional classroom methods, and the learners in these studies reported positive attitudes about e-learning with a high level of efficacy and acceptability by the operators and students [15].

This study demonstrated that YouTube videos can be an effective supplementary tool in periodontology education, showing significant knowledge improvement among students. The findings of the study provide strong evidence that YouTube is an effective supplementary tool for dental education. The variability in content quality highlights the importance of guidance from educators to ensure students access reliable and accurate resources. By integrating YouTube into the formal curriculum and encouraging academic participation in content creation, educators can maximize the platform’s potential while addressing its limitations.

Limitations and future perspective

Our study focused solely on final-year BDS undergraduate students from a single institution, which may limit the generalizability of the findings. Future research should include postgraduate students and a more diverse sample of institutions to provide a broader understanding of YouTube’s role in dental education. Further studies with larger sample sizes, diverse geographical locations, and populations are recommended to confirm these findings and explore the long-term benefits of using YouTube as a core part of dental education. A comparative study can be undertaken to assess the effectiveness of adjunctive use of YouTube videos with face-to-face teaching vs. face-to-face teaching alone. In addition, the creation of standardized, high-quality video content curated by educational institutions could further improve the effectiveness of YouTube as a teaching tool.

## Conclusions

Our study demonstrates that YouTube is an effective and well-received adjunctive educational tool for dental students, particularly in the field of periodontology. The findings reveal high levels of student satisfaction, engagement, and significant knowledge gain, with a mean difference in pre- and post-test scores of 18.90 ± 14.45 (P<0.0001). These results highlight the potential of integrating YouTube videos into the dental curriculum to enhance the overall learning experience. To maximize the educational value of YouTube, it is essential for faculty to curate reliable, evidence-based video content and to equip students with the skills needed to critically assess online resources. While YouTube has proven to be a valuable supplementary tool, its role should remain complementary to traditional teaching methods, ensuring a balanced and comprehensive educational approach. This study provides a foundation for future research on the use of digital platforms in dental education. It underscores the importance of integrating multimedia resources into modern teaching methodologies to meet the evolving needs of students and enhance educational outcomes in dentistry.

## Appendices

**Table 4:** Questionnaire used in the pre- and post-test.

Questionnaire
1) What are the different types of bone graft materials used in dental procedures? a) Metal and plastic b) Synthetic and natural c) Ceramic and glass d) Allograft, xenograft, and Alloplast
2) What are the properties of bone grafts? a) Osteogenesis b) Osteoconductive a) Osteogenesis d) All of the above
3) Example of autogenous bone graft? a) Osseous coagulum b) Bone blend c) Cancellous bone d) All of the above
4) Osseous coagulum is a mixture of? a) Bone and saline b) Bone dust and saline c) Bone and blood d) Bone dust and blood
5) What are the advantages of osseous coagulum? a) Ease of collection b) Adequate surface area c) None of the above d) Both A and B
6) Which type of bone graft is synthetic? a) Autograft b) Allograft c) Xenograft d) Alloplast
7) Which bone graft material is sourced from animals? a) Autograft b) Allograft c) Xenograft d) Alloplast
8) Which type of bone graft material is derived from the patient’s own body? a) Autograft b) Allograft c) Xenograft d) Alloplast
9) Which type of bone graft material is most likely to integrate with the patient’s existing bone? a) Alloplast b) Allograft c) Xenograft d) Autograft
10) What is bone graft material used for? a) To whiten teeth b) To replace missing teeth c) To replace the bone lost d) To clean teeth
11) What factors determine the success of a bone graft? a) Type of graft material b) Patient’s overall health c) Surgical technique and post-operative care d) All of the above
12) What are dental implants? a) Silver screw that integrates with the bone b) Titanium screw that integrates with the bone c) Silver + Titanium that integrate with the bone d) All of the above
13) The piece that connects the implants to the crown is called a) Cover screw b) Abutment c) Hex d) All of the above
14) The most important factors when considering dental implants a) Bone volume b) Bone quality c) Both a and b d) None of the above
15) Which bone is more dense? a) Maxilla b) Mandible c) Both 'a' and 'b' d) None of the above
16) The width and height of the bone can be increased by a procedure called a) Bone swaging b) Bone graft c) Both 'a' and 'b' d) None of the above
17) What is the primary function of the crown in a dental implant? a) To act as the visible part of the tooth b) To hold the implant in place c) To connect the implant post to the jawbone d) To clean the teeth
18) Which imaging technique is best used to plan dental implant placement? a) X-ray b) MRI c) CBCT scan d) Ultrasound
19) A surgical guide is used for- a) Placement of the implants b) To check the height of implants c) To measure the bone width d) All of the above
20) How does smoking affect dental implants? a) No effect b) Increases the risk of implant failure c) Improves osseointegration d) Enhances implant stability
21) What is a periodontal flap? a) Section of bone b) Section of mucosa c) Section of gingiva d) Both b and c
22) Classification of periodontal flap according to exposure of bone after reflection a) Full-thickness flap b) Partial thickness flap c) Both a and b d) None of the above
23) Full-thickness flap is also called as-a) Mucoperiosteal flap b) Mucosal flap c) Split-thickness flap d) Both a and b
24) Full-thickness flap is indicated in- a) Ressective surgery b) Regenerative surgery c) Both a and b d) None of the above
25) Partial thickness flap is also called as- a) Split-thickness flap b) Mucoperiosteal flap c) Both 'a' and 'b' d) All of the above
26) Partial thickness flap will not include-a) Connective tissue b) Periosteum c) Epithelium d) Connective tissue
27) A partial thickness flap is indicated in-a) Fenestration b) Dehiscence c) Apically positioned flap d) All of the above
28) Partial thickness flap is contraindicated in-a) When the gingiva is very thin > 1 mm b) Shallow vestibule c) Both 'a' and 'b' d) None of the above
29) Flap placement after surgery is classified into-a) Non-displaced flap b) Displaced flap c) Both a and b d) None of the above
30) Depending on the management of papilla flaps are classified into-a) Conventional b) Papilla preservation c) Both a and b d) None of the above
